# Antagonistic effect of protein extracts from *Streptococcus sanguinis* on pathogenic bacteria and fungi of the oral cavity

**DOI:** 10.3892/etm.2014.1618

**Published:** 2014-03-12

**Authors:** SHENGLI MA, HUI LI, CHUANG YAN, DAN WANG, HAIQING LI, XUE XIA, XUE DONG, YINGNAN ZHAO, TINGTING SUN, PENGFEI HU, WEIJUN GUAN

**Affiliations:** 1 Department of Stomatology, Hospital of Heilongjiang Province, Harbin, Heilongjiang 150036, P.R. China; 2 Institute of Animal Sciences, Chinese Academy of Agricultural Sciences, Beijing 100193, P.R. China

**Keywords:** *Streptococcus sanguinis*, intracellular proteins, exocrine proteins, antagonistic effect, bacteria, fungi

## Abstract

An antibacterial substance from *Streptococcus sanguinis* (*S. sanguinis*) is known to have an inhibitory effect on putative periodontal pathogens, but its inhibitory effect on pathogens of oral candidiasis is unknown. In this study, intracellular and exocrine proteins were extracted from *S. sanguinis*. The antagonistic effect of the protein extracts on *Prevotella intermedia* (*P. intermedia*) and *Porphyromonas gingivalis* (*P. gingivalis*) was detected by a well-plate technique, and the effects of the protein extracts on biofilms formed by these bacteria were evaluated by confocal laser scanning microscopy. The antagonistic effect of the protein extracts on pathogenic fungi was investigated using *Candida albicans* (*C. albicans*) and *Candida tropicalis* (*C. tropicalis*). The growth curves of *C. albicans* and *C. tropicalis* were determined from ultraviolet absorption measurements, their morphological changes following treatment were observed by optical microscopy and scanning electron microscopy, and the effects of the protein extracts on the thickness of their biofilms and the distribution of dead/live bacteria within the biofilms were detected by confocal laser scanning microscopy. The results showed significant inhibitory effects of the intracellular proteins extracted from *S. sanguinis* on pathogenic bacteria (*P. intermedia* and *P. gingivalis*), fungi (*C. albicans* and *C. tropicalis*) and the biofilms formed by them. Furthermore, the growth curves and morphology of *C. albicans* and *C. tropicalis* were altered following treatment with the intracellular proteins, resulting in disc-like depressions in the surfaces of the fungal spores and mycelia. By contrast, the exocrine proteins demonstrated no significant inhibitory effect on the pathogenic bacteria, fungi and the biofilms formed by them. Thus, it may be concluded that intracellular proteins of *S. sanguinis* have antibacterial activity and exert an antagonistic effect on certain pathogenic bacteria and fungi of the oral cavity.

## Introduction

*Streptococcus sanguinis* (*S. sanguinis*) is the dominant bacteria in a healthy oral cavity and it is generally accepted that an antibacterial substance generated by *S. sanguinis* has an inhibitory effect on putative periodontal pathogens. The main mechanism of this effect is the generation of hydrogen peroxide and bacteriocin, and the latter mainly functions under anaerobic conditions (1,2). It has been reported that bacteriocin accumulates in bacterial cells when *S. sanguinis* is cultured under anaerobic conditions, and is not released into the extracellular environment (3,4). However, antibacterial substances were detected in the culture medium of *S. sanguinis* in one particular study (5). The inhibitory effect of the antibacterial substance from *S. sanguinis* on the pathogens of oral candidiasis is unknown.

A previous study showed that there are considerable quantities of *Prevotella intermedia* (*P. intermedia*) and *Porphyromonas gingivalis* (*P. gingivalis*) in the subgingival plaque of adult patients with moderate or serious periodontitis (6). These two bacteria adhere and colonize under the gingiva. Disorganization of the periodontal supporting tissue and immune response of the body are initiated by these virulence factors, which play an important role in the development of periodontal diseases. Numerous studies have focused on investigating the biological functions of *P. intermedia* and *P. gingivalis* and elucidating their mechanisms of pathogenesis (7–10).

*Candida* is a common pathogenic yeast that induces deep fungal infections (11). The infection rate of *Candida* has increased greatly with the wide use of antibiotics, hormones and immune agents (12). A mixed infection of multiple *Candida* species is frequently detected in the oral cavity of patients with AIDS. The most common type of mixed infection is reported to be *Candida albicans* (*C. albicans*) complicated by *Candida tropicalis* (*C. tropicalis*), and *C. albicans* complicated by *Candida glabrata* or *Candida krusei* has also been observed (13), with *C. albicans* and *C. tropicalis* having the highest pathogenicity.

In the present study, intracellular and exocrine proteins were extracted from *S. sanguinis* and, using *P. intermedia*, *P. gingivalis*, *C. albicans* and *C. tropicalis* as indicators, the biological effects of the protein extracts on pathogenic bacteria and fungi were detected and analyzed.

## Materials and methods

### Extraction and purification of the intracellular and exocrine proteins from *S. sanguinis*

The standard strain ATCC 10556 of *S. sanguinis* was purchased from the State Key Laboratory of Oral Diseases, West China College of Stomatology, Sichuan University (Chengdu, China). Following identification and pure culture, the bacteria were inoculated on Brain Heart Infusion (BHI) culture medium and anaerobically cultured for 48 h.

### Intracellular proteins

The medium of *S. sanguinis* was ultracentrifuged at a low temperature following the anaerobic culture. The bacterial precipitate was collected, washed and resuspended in phosphate-buffered saline (PBS). The target proteins were released from the cells by sonication and the supernatant was collected by centrifugation (12,000 × g, 4°C, 30 min). Solid ammonium sulfate was slowly added to the supernatant to yield 60% saturation, salted out for 6 h at 4°C and centrifuged to isolate the supernatant. Subsequently, the collections were subjected to a second round of salting out with 70% saturation and, following centrifugation, the precipitate was dissolved in PBS. The desalting purification was conducted by chromatography on Sephadex G-25 (Pharmacia, Picastaway, NJ, USA). The collected materials were dialyzed, condensed, lyophilized and cryopreserved.

### Exocrine proteins

The medium of *S. sanguinis* was ultracentrifuged (10,000 × g, 4°C, 10 min) following the anaerobic culture. The supernatant was collected and an equal volume of cold anhydrous ethanol was added. The mixture was allowed to stand for 6 h at 4°C and then centrifuged. The precipitate was resuspended in PBS and cryopreserved.

### *P. intermedia* and *P. gingivalis*

The standard strains ATCC 25611 and ATCC 33277 of *P. intermedia* and *P. gingivalis*, respectively, were purchased from Beijing Stomatological Hospital, Capital Medical University (Beijing, China). The purified and identified *P. intermedia* and *P. gingivalis* were diluted to 1×10^8^ cfu/ml and a mixture of *P. intermedia* and *P. gingivalis* (ratio 1:1) was also prepared.

### Minimal inhibitory concentrations (MICs) of the protein extracts against *P. intermedia* and *P. gingivalis*

The MICs were detected by a well-plate technique. *P. intermedia* suspension (20 μl) was placed on a blood agar plate, spread evenly and two holes were punched with an aseptic puncher. The diameter of the holes was 5 mm. The agar in the hole was removed and sealed with melted BHI solid medium on the bottom (half the depth). Subsequently, 20 μl intracellular or exocrine *S. sanguinis* proteins were added to the holes and anaerobically cultured for 48 h at 37°C. The *P. gingivalis* and *P. intermedia* + *P. gingivalis* suspensions were treated by the same method. The MICs of the intracellular *S. sanguinis* proteins were determined by a double dilution method. The proteins were diluted to 0.5, 0.25, 0.125, 0.0625, 0.0313 and 0.0156 g/l with PBS. Subsequently, 20 μl dilutions to the equal volume of bacterial suspensions were obtained. Following complete mixing, 20 μl suspensions were spread evenly on the blood agar plates and anaerobically cultured for 48 h at 37°C. The MIC of the intracellular proteins of *S. sanguinis* against the *P. intermedia* and *P. gingivalis* co-culture was observed and calculated.

### Effects of the *S. sanguinis* proteins on biofilms formed by *P. intermedia* and *P. gingivalis*

A sterile cover glass with 500 μl *P. intermedia* and *P. gingivalis* mixture was placed into a six-well plate, allowed to stand for 10 min and then 2 ml BHI culture medium was slowly added and the mixture was anaerobically cultured for 48 h at 37°C to ensure that the planktonic *P. intermedia* and *P. gingivalis* were attached. Intracellular proteins (1 g/l) were slowly added to one well of the plate, exocrine proteins were slowly added to another well, and an equal volume of deionized water was added to the last well. The liquid from each well was carefully removed after 1 h treatment and the wells were washed with 1 ml PBS. Acridine orange (AO) and ethidium bromide (EB) were diluted to 100 mg/l with PBS, the dilutions were mixed and re-diluted 20-fold, added to the wells, and observed under a confocal laser scanning microscope (Leica CTR6500; Leica, Wetzlar, Germany). When the live bacterial DNA combined with AO, bright green fluorescence was observed, and when the dead bacterial DNA combined with EB, red fluorescence was observed. Yellow or orange fluorescence indicated the overlap of live and dead bacteria. Biofilm viability (BV; %) = green fluorescence/(green fluorescence + red fluorescence) × 100.

### C. albicans and C. tropicalis

The standard strains ATCC 10231 and ATCC 13803 of *C. albicans* and *C. tropicalis*, respectively, were purchased from the National Center for Medical Culture Collections (Beijing, China). The purified and identified *C. albicans* and *C. tropicalis* were diluted to 1×10^6^ cfu/ml with RPMI-1640 culture medium, and a mixture of *C. albicans* and *C. tropicalis* (ratio 1:1) was also prepared.

### MICs of the protein extracts against C. albicans and C. tropicalis

The MICs of the *S. sanguinis* protein extracts against *C. albicans*, *C. tropicalis* and their co-cultures were detected using a well-plate technique. The intracellular and exocrine proteins of *S. sanguinis* were diluted to 0.25, 0.5 and 1 g/l with PBS. The tests were performed on three groups. In the first group, *C. albicans* suspension (10 μl) was inoculated into a culture tube with 1 ml RPMI-1640 and 300 μl intracellular proteins. In the second group, *C. albicans* suspension (10 μl) was inoculated into a culture tube with 1 ml RPMI-1640 and 300 μl exocrine proteins. Group three was used as a control without any protein extracts. The test groups were cultured at 37°C with agitation for 12 h; then they were sampled and the bacterial culture was counted. The MICs of the *S. sanguinis* protein extracts against the *C. tropicalis* culture and *C. albicans* and *C. tropicalis* co-culture were observed and calculated using the same method.

### Effects of the S. sanguinis proteins on biofilms formed by C. albicans and C. tropicalis

The biofilm models of *C. albicans* and *C. tropicalis* were established by the same method as that described for *P. intermedia* and *P. gingivalis*. The effects of the intracellular and exocrine proteins on the biofilm models were tested, 500 μl proteins (1 g/l) were added, 500 μl 5.25% chlorhexidine was used as positive control, two techniques were used: Continuous tomography to determine the thickness of the biofilms, and dynamic observation of adherent viable/dead bacteria in the different periods of biofilm formation.

### Effects of the intracellular proteins on the growth of C. albicans and C. tropicalis

The tests were performed on two groups (Table I). Each group was cultured at 37°C and agitated, and the optical density (OD) value at 600 nm was tested every 2 h. Growth curves (y-axis, OD value and x-axis, incubation time) were plotted to assess the proliferation ability of each group.

### Effects of the intracellular proteins on the morphology of C. albicans and C. tropicalis

The tests were performed on two groups (Table I). Each group was cultured at 37°C and agitated for 12 h and histological examination was conducted to observe the morphology by optical microscopy (OM; SH11/YF-9, Shenzhen Xingyu Xin Electronics Co., Ltd., Guangdong, China) and scanning electron microscopy (SEM; Magellan XHR, FEI, USA).

### Statistics and analysis

X-test, one-way analysis of variance and logistic analysis were conducted using SPSS 16.0 to make statistical analysis. P<0.05 was considered to indicate a statically significant result.

## Results

### Effects of the protein extracts on *P. intermedia* and *P. gingivalis* Inhibitory effect

A marked inhibitory effect of the intracellular proteins on the growth of *P. intermedia* and *P. gingivalis* was observed, while the exocrine proteins appeared to be inactive. The MIC of the intracellular proteins against the co-culture of *P. intermedia* and *P. gingivalis* was 0.125 g/l (Table II).

### Results tested by confocal laser scanning microscopy

**Figure 1:**
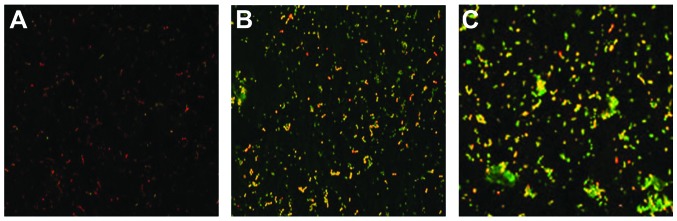
Distribution of the dead/viable bacteria in the biofilms formed by a *P. intermedia* and *P. gingivalis* co-culture observed by confocal laser scanning microscopy. Acridine orange (AO) and ethidium bromide (EB) staining; magnification, ×200. (A) Intracellular proteins of *S. sanguinis*; (B) exocrine proteins of *S. sanguinis*; and (C) deionized water. *P. intermedia, Prevotella intermedia; P. gingivalis, Porphyromonas gingivalis; S. sanguinis, Streptococcus sanguinis.*

A significant reduction in the percentage of viable cells in the *P. intermedia* and *P. gingivalis* co-cultural biofilm following treatment with the intracellular proteins was observed compared with that of the control biofilm (P<0.05), while no significant change following treatment with the exocrine proteins was identified compared with that of the control group (Table III and Fig. 1).

### Effects of the protein extracts on C. albicans and C. tropicalis Inhibitory effect

A significant inhibitory effect of the intracellular proteins on the growth of *C. albicans* and *C. tropicalis* was observed compared with that of the control group, while the exocrine proteins did not have a significant effect. When the concentration of the intracellular proteins was 1 g/l, the number of fungal colonies was significantly different between the *C. albicans*, *C. tropicalis* and their co-culture groups and the corresponding control group (Table IV).

### Results tested by confocal laser scanning microscopy

The minimum biofilm thickness of *C. albicans* and *C. tropicalis* was achieved within 12 h in the intracellular protein groups. The thickness gradually reduced between 4 and 12 h, and then increased between 12 and 48 h (Tables V and VI).

**Figure 2:**
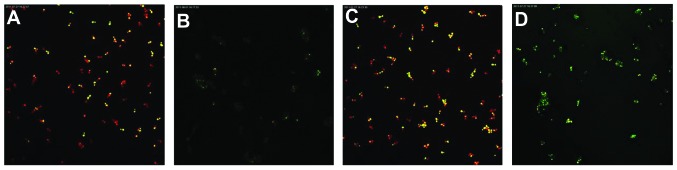
Distribution of dead/live fungi within the *C. albicans* biofilm shown by fluorescence analysis. Acridine orange (AO) and ethidium bromide (EB) staining; magnification, ×200. (A) Intracellular protein, 12 h; (B) exocrine protein, 12 h; (C) chlorhexidine, 12 h; and (D) deionized water, 12 h. *C. albicans*, *Candida albicans.*

**Figure 3:**
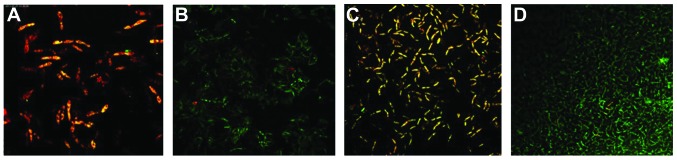
Distribution of dead/live fungi within the *C. tropicalis* biofilm shown by fluorescence analysis. Acridine orange (AO) and ethidium bromide (EB) staining; magnification, ×200. (A) Intracellular protein, 12 h; (B) exocrine protein, 12 h; (C) chlorhexidine, 12 h; and (D) deionized water, 12 h. *C. tropicalis, Candida tropicalis.*

Following fluorescent staining, it was identified that there were a number of dead fungi present at the bottom of the biofilm formed by the *C. albicans* and *C. tropicalis* co-culture. The intermediate layer was a mixture of live and dead fungi, while there were few fungi in the surface layer. The quantity of live *C. albicans* and *C. tropicalis* following treatment with the intracellular proteins was less than that of the control group for 4–12 h, while no significant difference was observed after 24–48 h. The quantity of live *C. albicans* and *C. tropicalis* following treatment with the exocrine proteins was fundamentally the same as that of the control group (Figs. 2 and 3).

### Growth curve

**Figure 4:**
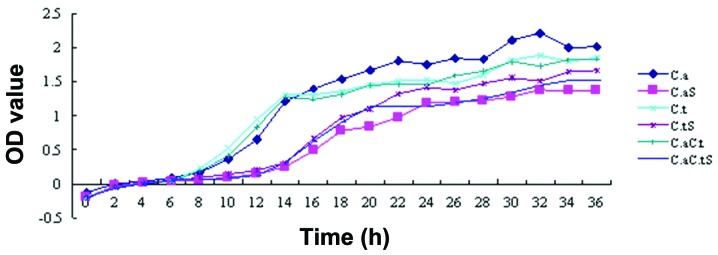
Effect of the intracellular proteins of *Streptococcus sanguinis* on the growth curves of *Candida albicans* (C.a), *Candida tropicalis* (C.t) and their co-culture (C.sC.t). C.a, C.t and C.aC.t are control cultures and C.aS, C.tS and C.aC.tS are the corresponding protein-treated cultures. OD, optical density.

The OD values represent the radiation absorbed by a detected object. The turbidity of a bacterial suspension is proportional to the OD, which is precisely measured by an ultraviolet spectrophotometer; therefore, OD values represent the relative quantity of bacteria under a certain experimental condition, thus reflecting the relative amount of growth. The results showed a lag phase growth of *C. albicans*, *C. tropicalis* and their co-culture following treatment with the intracellular proteins for 14 h after seeding, after which the bacteria proliferated rapidly and entered a logarithmic phase, while the control groups entered this phase at 6 h. It was indicated that the growth of *C. albicans*, *C. tropicalis* and their co-culture was inhibited by the intracellular proteins of *S. sanguinis*; the inhibitory effect began after 8 h of incubation and was no longer observed at 14 h (Fig. 4).

### Morphological observation

**Figure 5:**
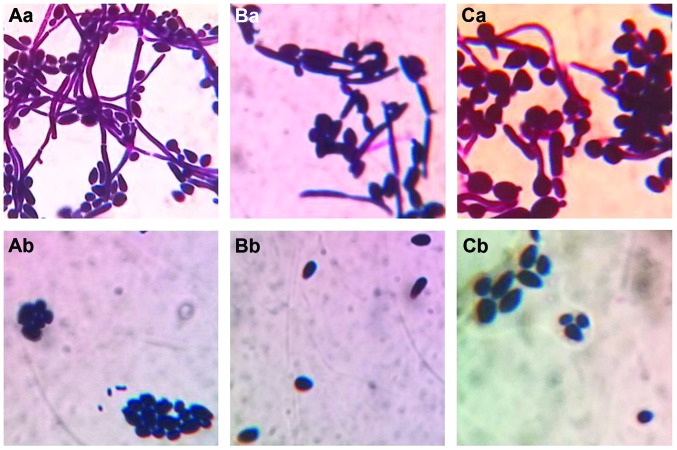
Morphology of *C. albicans* and *C. tropicalis* in the control and the test groups under OM observation. (Aa) *C. albicans*, 12 h; (Ab) intracellular protein-treated *C. albicans*, 12 h. (Ba) *C. tropicalis*, 12 h; (Bb) intracellular protein-treated *C. tropicalis*, 12 h. (Ca) *C. albicans* and *C. tropicalis* co-culture, 12 h; (Cb) intracellular protein-treated co-culture, 12 h. Magnification, ×1,000. *C. albicans, Candida albicans; C. tropicalis, Candida tropicalis;* OM, optical microscopy.

Using OM, it was observed that there was a large number of spores and hyphae in the control group culture of *C. albicans*, *C. tropicalis* or their co-culture. The hyphae were different in length, and segmentally and irregularly distributed among round or oval spores. When treated with the intracellular proteins, the number of spores was markedly reduced, and there was no hypae growth among round or oval spores (Fig. 5).

**Figure 6:**
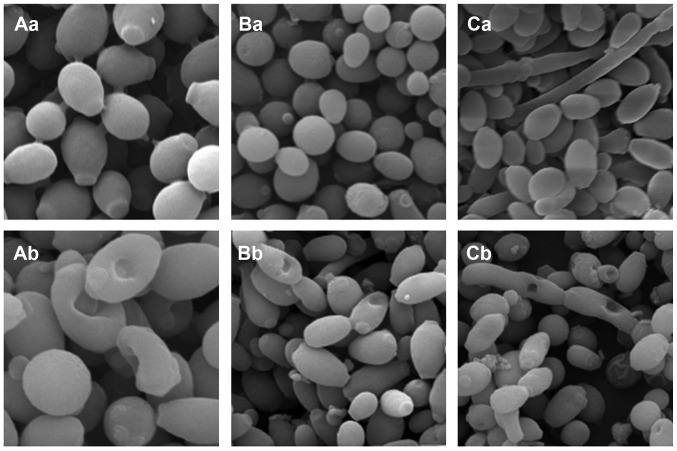
Morphology of *C. albicans* and *C. tropicalis* in the control and the test groups under SEM observation. (Aa) *C. albicans*, 12 h; (Ab) intracellular protein-*C. albicans*, 12 h (magnification, ×4,000). (Ba) *C. tropicalis*, 12 h; (Bb) intracellular protein-*C. tropicalis*, 12 h (magnification, ×2,000). (Ca) *C. albicans* and *C. tropicalis* co-cultures, 12 h; (Cb) intracellular protein-co-cultures, 12 h (magnification, ×2,000). *C. albicans, Candida albicans; C. tropicalis, Candida tropicalis;* SEM, scanning electron microscopy.

Using SEM, it was identified that there were round or oval spores in the control group cultures of *C. albicans* and *C. tropicalis*; the spores were 2–5 μm in diameter, had a full shape and smooth surface, and there were adhesions among a number of the spores. Round or oval spores and segmental hyphae were observed in the *C. albicans* and *C. tropicalis* co-culture control group; the spores had a full shape and smooth surface, the spore diameter was 2–5 μm, and the hyphae took up the entire photograph with a diameter of 1–3 μm. When treated with the intracellular proteins, certain oblate or irregular spores were identified in the *C. albicans* and *C. tropicalis* monoculture groups. Disc-like depressions of different depths were present in the surfaces of the spores, and the diameter of the depressions was 0.2–0.8 μm. The majority of the spores had only one depression, while their surfaces were smooth and free of wrinkles. In the intracellular protein-treated *C. albicans* and *C. tropicalis* co-culture group, disc-like depressions were observed in the surfaces of a number of spores, and the diameter of the depressions was 0.2–0.8 μm. The majority of the spores had only one depression, although two depressions were occasionally observed. Depressions were also identified in the surface of the segmental hyphae. Each segment had a depression, the diameter of the hyphae was 2 μm and that of the depression was 0.6 μm. The surfaces of the hyphae and the majority of the spores were smooth; however, wrinkles were present in the surfaces of certain spores (Fig. 6).

## Discussion

It is controversial whether the antibacterial substance generated by *S. sanguinis* under anaerobic conditions exists in the cell or is released into the extracellular environment. In this study, intracellular and exocrine proteins were extracted from *S. sanguinis*, and their antagonistic effects on *P. intermedia*, *P. gingivalis*, *C. albicans* and *C. tropicalis* were detected. The results showed that the antibacterial substance of *S. sanguinis* was obtainable through a series of methods, namely centrifugation, ultrasonication, salting out, Sephadex G-50 filtration and dialysis. The extracts are also known as bacteriocins. The putative periodontal pathogens *P. intermedia* and *P. gingivalis* are bacteria, while *C. albicans* and *C. tropicalis* are fungi, so they are discussed separately in this study.

In the present study, a number of effects of the protein extracts on *P. intermedia* and *P. gingivalis* were observed. *P. intermedia* is a Gram-negative anaerobic bacteria that produces melanin, and is a potentially pathogenic bacteria that is frequently detected in the subgingival plaques of patients with periodontitis, pregnancy gingivitis, acute necrotizing gingivitis and human immunodeficiency virus-associated gingivitis (14). *P. gingivalis* is considered as another main periodontal pathogen, and is associated with adult periodontitis, juvenile periodontitis, periodontal abscesses, alveolar abscesses, pulp infection and refractory periodontitis (15). *P. intermedia* and *P. gingivalis* are two important putative periodontal pathogens, and their virulence factors penetrate and destroy the host tissue, allowing them to escape the host defense system. The detection rate is high in deep periodontal pockets and the attachment loss sites of periodontitis. It has been reported that the development of periodontitis is severely associated with *P. gingivalis* and moderately associated with *P. intermedia* (16). Biofilms are the main form of survival for pathogens; they adapt themselves to the circumstances and resist attack from phagocytes, and inflammatory factors released by them cause the local inflammatory response. Complicated biofilms are the main pattern of existence of putative periodontal pathogens, and adhesive interactions between the bacteria form barriers that develop resistance to antibiotics. The results of the present study showed a marked effect of the intracellular proteins on the growth of *P. intermedia*, *P. gingivalis* and their co-cultures. Also, the biofilm viability of the *P. intermedia* and *P. gingivalis* co-culture was significantly reduced following treatment with the intracellular proteins, compared with that of the control group. It may speculated that: i) Secretory immunoglobulin A (SIgA) in the saliva plays a pivotal role in the local anti-infective immune response of the oral mucosa. SIgA may bind specifically to antigens and play an inhibitory role in sterilization through a biological effect on bacteria. As the intracellular proteins of *S. sanguinis* interacted with the biofilm formed by the *P. intermedia* and *P. gingivalis* co-culture and the viability of the biofilm was reduced, it was considered that the mechanisms of action of SIgA and *S. sanguinis* may be similar. ii) Certain small molecules of the intracellular proteins of *S. sanguinis* may act as ligands that specifically bind to cell-membrane or intracellular receptors of *P. intermedia* and *P. gingivalis*, and then trigger a cascade of cellular events.

In the present study, a significant inhibitory effect of the intracellular proteins on the growth of *C. albicans*, *C. tropicalis* and their co-culture was observed compared with that of the control groups. In clinical practice, the commonly used antifungal drugs mainly function through the inhibition of ergosterol biosynthesis, and as ergosterol is the major component of the fungal cell membrane, fungal growth is inhibited (17,18). To investigate whether the inhibitory mechanism of the intracellular proteins of *S. sanguinis* on *C. albicans* and *C. tropicalis* was consistent with the mechanism of typical antifungal drugs, in the present study, the effects of the proteins of *S. sanguinis* on the morphology of *C. albicans* and *C. tropicalis* were analyzed. It was demonstrated that the number of spores and hyphae of *C. albicans* and *C. tropicalis* was markedly reduced following treatment with the intracellular proteins, and that disc-like depressions were present in the surfaces of spores and hyphae. Combined with the MIC values, the conclusion that the intracellular proteins of *S. sanguinis* not only caused the morphological changes of *C. albicans* and *C. tropicalis*, but also the inhibition of their growth was reached. There may be a number of associations between them, so it is considered that the intracellular proteins of *S. sanguinis* function through the following mechanisms: i) Numerous intracellular proteins assemble on the cell surface by electrostatic forces, which leads to a change in the distribution of certain molecules in the outer layer of the cell and eventually to a change in the morphology of the cell. ii) Intracellular proteins may specifically bind to certain substances of the cell membrane or walls of *C. albicans* and *C. tropicalis*, to cause the breakdown of the outer layer. Wiedemann *et al* (19) and Christ *et al* (20) showed that the primary force of the antibacterial effect of the bacteriocin of lactic acid bacteria is the formation of pores of diameter 2–2.5 nm on the surface of target cells, leading to an increase in cell membrane permeability, which allows the release of ATP, amino acids and ions from the cell. This results in bacterial metabolic disturbances and ultimately causes the death of the cell. These morphological changes are accordant with the results generated by the intracellular proteins of *S. sanguinis* in the present study, so it may be speculated that the antifungal effect was the result of a loss of integrity of the cell surface and changes in cell membrane permeability. Whether the intracellular proteins of *S. sanguinis* are able to enter cells and influence signal transduction and gene expression requires further study. iii) A certain type of enzyme may be released from *S. sanguinis*, which interacts with the cell walls through electrostatic attraction, and then the cell is depressed and lysosomal enzyme is released, which induces autocytolysis. iv) The intracellular proteins may enter the cell and inhibit the synthesis of chromosomal DNA, releasing the cytoskeleton and thereby inducing autocytolysis. v) The intracellular proteins may enter the cell and inhibit the synthesis of chromosomal DNA; this is likely to damage the cytoskeleton to a certain extent, and ultimately result in cytoskeleton collapse and the formation of disc-like depressions on the bacterial surface.

The results of the growth curve analysis in the present study indicated that the growth of *C. albicans*, *C. tropicalis* and their co-culture was inhibited by the intracellular proteins of *S. sanguinis* at a concentration of 1 g/l; the inhibition began at 8 h and ceased at 14 h. The reasons for this observation may be that: i) There may be a *‘*half-life period’ of the intracellular proteins of *S. sanguinis*; that is, they are degraded by fungi or fungal metabolites as the treatment time is extended. ii) The inhibition process of the intracellular proteins of *S. sanguinis* may be a form of active transport; the intracellular proteins are transported by an active transcellular route against the concentration gradient, and as the process requires energy expenditure, the inhibitory effect reduces gradually.

The thickness of the biofilms formed by *C. albicans* and *C. tropicalis* was reduced following treatment with the intracellular proteins within 24 h and minimized in 12 h, suggesting the intracellular proteins of *S. sanguinis* have significant antifungal effects. It may be speculated that: i) The antifungal substance of *S. sanguinis* is located within the cell. ii) The intracellular proteins are gradually degraded by fungi or fungal metabolites, their *‘*half-life period’ is minimized and biological activity is lost. iii) The intracellular proteins are characterized by amphoteric dissociation; when they become positively charged, the negative charges of *C. albicans* or *C. tropicalis* are neutralized, which triggers a cascade of cellular potential disorders and finally causes the death of the bacteria. iv) The intracellular proteins may cause changes in the osmotic balance of *C. tropicalis in vitro* and *in vivo*; as the proteins are gradually degraded through facilitated diffusion within 24 h, the antifungal effect is then reduced. v) The van der Waals force between the bacteria increases along with the maturation of the biofilm, which is tightly packed, so the antifungal substance is not able to enter the biofilm and the inhibitory effect is invalidated, with little effect on the bacterial surface.

No inhibitory effect of the exocrine proteins on the growth of *P. intermedia*, *P. gingivalis*, *C. albicans* and *C. tropicalis* was observed in the present study, which is not accordant with the findings of previous studies (21). The standard strain of *S. sanguinis* or the culture conditions in the present study may have been different from those of the other studies, resulting in the antimicrobial substance of *S. sanguinis* not being exported out of the cell in the previous studies.

In the present study, significant inhibitory effects of the intracellular proteins of *S. sanguinis* on the growth of *P. intermedia*, *P. gingivalis*, *C. albicans*, *C. tropicalis* and their biofilms were observed, and the morphology of *C. albicans* and *C. tropicalis* was also affected. However the inhibitory mechanism of *S. sanguinis* was not identified. Whether the inhibition caused the morphological changes or the morphological changes caused the inhibition, or they were essential prerequisites of each other was not fully elucidated. Furthermore, identification of where the antimicrobial substance of *S. sanguinis* is located requires further study.

## Figures and Tables

**Table I:** Shorthand and substances of the groups in each experiment.

Group	Intracellular proteins 1 g/l	*C. albicans* suspension	*C. tropicalis* suspension	RPMI-1640 medium
Treatment				
Intracellular proteins *C. albicans*	300 μl	10 μl	-	10 ml
Intracellular proteins *C. tropicalis*	300 μl	-	10 μl	10 ml
Intracellular proteins co-cultures	300 μl	5 μl	5 μl	10 ml
Control				
*C. albicans*	-	10 μl	-	10 ml
*C. tropicalis*	-	-	10 μl	10 ml
*C. albicans* and *C. tropicalis*	-	5 μl	5 μl	10 ml

C. albicans, Candida albicans; C. tropicalis, Candida tropicalis.

**Table II:** Inhibitory effect of the bacterial intracellular and exocrine proteins of *S. sanguinis* on *P. intermedia* and *P. gingivalis*.

Bacteria	Intracellular proteins	MIC (g/l)	Exocrine proteins
*P. intermedia*	+	0.1250	−
*P. gingivalis*	+	0.0625	−
*P. intermedia* + *P. gingivalis*	+	0.1250	−

‘+’ represents inhibition of bacterial growth, ‘−’ represents no inhibition of bacterial growth. *P. intermedia*, *Prevotella intermedia; P. gingivalis*, *Porphyromonas gingivalis; S. sanguinis*, *Streptococcus sanguinis*; MIC, minimal inhibitory concentration of the intracellular proteins.

**Table III:** Effects of the intracellular and exocrine proteins of *S. sanguinis* on the viability of the biofilms formed by the *P. intermedia* and *P. gingivalis* co-culture (%, mean ± standard deviation).

Measured Item	Intracellular proteins	Exocrine proteins	Deionized water
Biofilm Viability	28.33±3.87^a^	54.41±4.12	56.44±4.79

a Significant difference between the intracellular protein and control groups (P<0.05).

*S. sanguinis*, *Streptococcus sanguinis; P. intermedia*, *Prevotella intermedia; P. gingivalis*, *Porphyromonas gingivalis*.

**Table IV:** Inhibitory effect of the intracellular and exocrine proteins of *S. sanguinis* on *C. albicans* and *C. tropicalis* (10^6^ cfu/ml, mean ± standard deviation).

	Intracellular proteins (g/l)			Exocrine proteins (g/l)			
			
Fungi	0.25	0.5	1	0.25	0.5	1	Control group
*C. albicans*	1.95±0.26	1.86±0.31	0.72±0.13^a^	1.98±0.19	1.96±0.09	1.98±0.32	2.12±0.14
*C. tropicalis*	1.87±0.29	1.70±0.36	0.72±0.18^a^	2.13±0.21	1.97±1.03	1.83±0.28	2.07±0.15
*C. albicans + C. tropicalis*	1.85±0.18	1.70±0.34	0.69±0.13^a^	2.05±0.14	1.89±0.22	1.85±0.15	2.11±0.17

a Significant difference compared with the corresponding control (P<0.05).

*S. sanguinis*, *Streptococcus sanguinis; C. albicans, Candida albicans; C. tropicalis, Candida tropicalis.*

**Table V:** *C. albicans* biofilm thickness at different time points (μm, mean ± standard deviation).

	Time (h)				
	
Group	4	8	12	24	48
Intracellular proteins	33.03±1.87	25.55±2.05^a^	15.50±41.47^a^	30.59±1.85^a^	56.55±3.85
Exocrine proteins	33.28±2.15	41.53±2.13	42.24±2.26	73.19±2.21	94.03±2.66
Chlorhexidine	35.46±1.92	31.55±2.59	26.12±1.29	24.50±1.76	37.38±3.56
Deionized water	34.37±2.11	40.97±1.22	41.11±2.08	71.20±2.52	96.96±3.60

a Significant difference between the intracellular protein and deionized water groups in the biofilm thickness at 8, 12 and 24 h (P<0.05).

C. albicans, Candida albicans.

**Table VI:** *C. tropicalis* biofilm thickness at different time points (μm, mean ± standard deviation).

	Time (h)				
	
Group	4	8	12	24	48
Intracellular proteins	35.33±5.74	33.91±4.58^a^	27.72±4.00^a^	37.69±3.57^a^	76.69±5.17
Exocrine proteins	33.28±2.15	47.36±2.79	62.36±4.46	75.18±4.60	103.04±6.55
Chlorhexidine	32.23±2.83	33.13±3.63	35.91±3.60	42.55±5.05	58.18±4.45
Deionized water	33.91±2.06	44.08±4.68	58.21±3.38	71.17±4.40	112.26±5.27

a Significant difference between the intracellular protein and deionized water groups in the biofilm thickness at 8, 12 and 24 h (P<0.05).

C. tropicalis, Candida tropicalis.
